# Enhancing vaccine immunogenicity through heterologous prime-boost regimen with multiple nasal boosting with liposomal TLR7 ligand

**DOI:** 10.1016/j.vaccine.2025.127778

**Published:** 2025-10-08

**Authors:** Tomoko Hayashi, Shiyin Yao, Fumi Sato-Kaneko, Renna Cozza, Hiroyuki Baba, Jasmine Jin, Ian Mclaughlin, Fernando Gil, Paola Anguiano Quiroz, Nikunj M. Shukla, Michael Chan, Howard B. Cottam, Dennis A. Carson

**Affiliations:** Division of Rheumatology, Department of Medicine, University of California San Diego, 9500 Gilman Dr, La Jolla, CA 92093-0809, United States

**Keywords:** Influenza virus vaccines, Liposomal formulation, Toll-like receptor 7, 1V270, Intranasal vaccine adjuvant

## Abstract

**Objectives:**

Despite the clinical efficacy of current intramuscular influenza vaccines in reducing the severity of seasonal infection, they exhibit limited induction of mucosal immunity, which is essential for preventing viral transmission. In addition, intranasal vaccination can induce superior mucosal immunity, enhancing clinical efficacy and reducing transmission, and its self-boosting potential may improve coverage in older adults and those with mobility limitations.

**Methods:**

We developed Lipo-**1V270**, a liposomal nanoparticle formulation of the synthetic TLR7 agonist **1V270** using 1,2-dioleoyl-sn-glycero-3-phosphocholine (DOPC) and cholesterol for mucosal vaccine delivery. In vitro immune stimulation and in vivo immunogenicity were evaluated using intramuscular and intranasal routes in mouse models, including a heterologous prime-boost regimen with inactivated influenza A virus [IIAV, A/California/04/2009 (H1N1)pdm09] adjuvanted monophosphoryl lipid A (MPLA) priming.

**Results:**

In vitro analysis showed that Lipo-**1V270** exhibited attenuated innate immune potency compared to unformulated **1V270**. However, in vivo co-administration of Lipo-**1V270** with IIAV significantly enhanced antigen-specific IgG1 and IgG2a responses. Subsequently, intranasal boosting with Lipo-**1V270**, following intramuscular priming with IIAV adjuvanted with MPLA - a component included in FDA-approved vaccines - elicited robust influenza-hemagglutinin (HA)-specific mucosal IgA and IgG responses in nasal wash. This heterologous prime-boost regimen also induced strong splenic T-cell responses and HA-specific IgG and IgA antibodies in nasal wash without causing significant weight loss for 7 days post-boost in immunized mice.

**Conclusions:**

Intranasal administration of Lipo-**1V270** in a heterologous prime-boost vaccination regimen effectively enhances mucosal immunity against influenza virus infection, with an acceptable innate immune-mediated adverse effects profile. This strategy may be applicable to vaccines against other respiratory infectious diseases.

## 1. Introduction

Intranasal vaccination delivery has emerged as a promising non-invasive method for vaccine administration, providing a convenient and effective approach to prevent respiratory infection [1]. The nasal route is approved by the Food and Drug Administration (FDA) for administration of live attenuated influenza type A and B vaccines [2]. Intranasal vaccination elicits both systemic and mucosal immunity, making it an ideal approach for mass immunization, including in elderly patients and in children [3]. This approach not only enhances immune responses but also prevents the transmission of respiratory pathogens by targeting the nasal mucosa, which is the first point of virus entry, before they reach the lungs. Furthermore, nasal administration is convenient, as it is easily accessible and does not require medically trained personnel, allowing for the potential for repeated dosing.

To enhance mucosal vaccine efficacy, nanoparticle-based delivery systems such as liposomes have been investigated to improve antigen stability, to facilitate uptake by antigen-presenting cells, and to promote slow release of vaccines. Liposomal formulations have shown clinical success in vaccines such as AS01B in Shingrix, an intramuscular subunit vaccine against herpes zoster [4]. In intranasal delivery, liposomal formulation improves bioavailability, targets the respiratory mucosal epithelium, prolongs localization for sustained release, and reduces systemic toxicity. This approach enhances the immunogenicity of vaccines, while also improving their safety and stability [1,5].

Toll-like receptors (TLRs) recognize pathogen-associated molecular patterns (PAMPs), and currently, 10 TLRs have been identified in humans and 13 in mice, respectively. In humans, TLR7 is located in the endosomal compartments in plasmacytoid dendritic cells (pDC) and B cells. In mice, TLR7 is also expressed in conventional dendritic cells, as well as myeloid cells, pDC and B cells [6,7]. Given that TLR7 is expressed in the endosomal compartment in limited immune cells and is unique in that its ligand can be a small-molecular-weight compound, we selected TLR7 ligands as an immunomodulator with easy-synthesis for further optimization [8,9].

Our laboratory previously identified a synthetic TLR7 ligand, **1V136** (SM360320, oxoadenine), and conducted an extensive structure-activity-relationship (SAR) study. We subsequently developed **1V209** as a versatile conjugatable TLR7 ligand and compared immunostimulatory activities of TLR7 ligands conjugated to various molecules, e.g., protein, polyethylene glycol, and lipids [10]. Briefly, **1V270** was obtained by chemical conjugation of carboxylic acid moiety of **1V209** to amino functional group of phospholipid; 1,2-dioleoyl-sn-glycero-3-phosphoethanolamine (DOPE) [10]. Thus, **1V270** is an amide-linked lipid-conjugated oxoadenine class of TLR7 ligand (Fig. S1). **1V270**, conjugated to DOPE demonstrated strong vaccine adjuvant activity in preclinical murine models. As a single agent, **1V270** enhanced the immunogenicity of protein antigens such as ovalbumin (OVA) administered intramuscularly or subcutaneously and also showed potent anti-cancer immunotherapeutic effects in a murine head and neck cancer model [10,11]. Previous studies have demonstrated that the synthetic TLR7 agonist **1V270** possesses minimal off-target effects and has a wide therapeutic window [12,13]. **1V270** activates the TLR7 pathway that requires the adaptor protein MYD88 [14].

We have also previously reported a heterologous prime-boosting vaccination regimen using intramuscular priming and intranasal boosting with inactivated influenza A virus [IIAV, A/California/04/2009/(H1N1)pdm09] adjuvanted with Fos47, which is a liposomal formulation containing **1V270** and our proprietary TLR4 ligand (**2B182C**) [15]. This regimen elicited robust immune responses in mice and was well-tolerated, as evidenced by the absence of weight loss during the 7-day period after intranasal boosting [15].

We build on those findings by evaluating the liposomal formulation of **1V270** (Lipo-**1V270**) as an adjuvant in combination with IIAV antigen. Here, we present a comprehensive evaluation of Lipo-**1V270** in both in vitro and in vivo models. In this study, we aim to assess its innate immune stimulatory capacity, characterize its adjuvant activity, using the vaccination regimen of intramuscular priming with IIAV adjuvant with MPLA, followed by multiple intranasal boosts of IIAV adjuvanted with Lipo-**1V270**. An extended five-dose schedule with repeated intranasal boosts was employed to evaluate the feasibility and immunological potential of multiple boosting with Lipo-**1V270.** These findings provide a foundation for further development of Lipo-**1V270** as an adjuvant for intranasal vaccination against respiratory infectious diseases.

## 2. Materials and methods

### 2.1. Preparation of Lipo-1V270

#### Instrumentation.

Organic solutions were dried for thin-film formation with a Büchi Rotavapor R-205 (New Castle, DE). Lipid nanoparticles were formed using both a Branson Bransonic^®^ M Mechanical Bath 2800 (Branson, Brookfield, CT) and a Fisherbrand^™^ Model 120 Sonic Dismembrator (Waltham, MA). Qualitative analyses were performed to verify the purity of the compound in the formulation using an Agilent 1260 LC/6420 Triple Quad mass spectrometer (Santa Clara, CA) with Onyx Monolithic C18 (Phenomenex, Torrance, CA) column.

#### Methods.

**1V270** was synthesized as previously described [10] (Fig. S1). More than ninety-nine % purity and 12 months (4 °C) of stability were determined by HPLC-MS (Fig. S3).

Lipid nanoparticle formulations of **1V270** were prepared via the thin-film rehydration method (Fig. S2). A mixture of **1V270**, 1,2-dioleoyl-sn-glycero-3-phosphocholine (DOPC, Avanti Polar Lipids, Alabaster, AL) and cholesterol (Avanti Polar Lipids, Alabaster, AL) at a molar ratio of 1:15:7.5 was solubilized in 9:1 volumetric ratio of chloroform: methanol and added to a vial. The organic solution was removed gently by rotary evaporator at 35–40 °C and further dried under vacuum overnight to form a uniform thin film. The thin film was rehydrated with the appropriate volume of 1× dPBS (Gibco, Waltham, MA) and sonicated in a 45 °C sonicating water bath for 15 min or until the thin film had fully detached from the walls of the vial. Following the sonicating bath rehydration, the suspension was further sonicated by a probe sonicator at 30 % power for 2 min in intervals of 15 s with 10-s rest periods on ice to prevent overheating of the sample. All lipid nanoparticles were formed using aseptic techniques using endotoxin-free consumables.

### 2.2. Animals

Female BALB/c and C57BL/6 mice (6–8 weeks old) were purchased from the Jackson Laboratory (Bar Harbor, ME, USA). BALB/c mice were used for in vivo immunization studies, while C57BL/6 mice were used for the preparation of murine bone marrow-derived dendritic cells (mBMDCs). Both strains are widely utilized in immunological research and exhibit distinct genetic backgrounds and immune response characteristics [16]. The mice were first acclimated for at least 48 h after arrival at a vivarium and then used for studies. All mouse experiments were performed at the University of California (UC) San Diego Animal Facility. The animal husbandry was performed by UC San Diego Animal Care Program, including 24-h veterinary care support.

### 2.3. Cell cultures of mBMDC and PBMC

Murine bone marrow-derived dendritic cells (mBMDCs) were prepared from bone marrow cells harvested from femurs and tibias of C57BL/6 or BALB/c mice as previously described [17]. Briefly, bone marrow cells from mice were cultured at 8 × 10^5^ cells/mL in RPMI-1640 medium supplemented with 10 % fetal bovine serum (Thermo Fisher Scientific) and 1 % penicillin/streptomycin (Thermo Fisher Scientific) (RP-10), 0.02 μg/mL GM-CSF (BioLegend). Medium was refreshed on days 3 and 6, and nonadherent cells were harvested on day 7. Prior to stimulation, cells were washed once with fresh RP-10 and adjusted to a final density of 1 × 10^5^ cells/well. mBMDC were then incubated with compound or vehicle (0.5 %, dimethyl sulfoxide, DMSO) in RP-10. Human peripheral blood mononuclear cells (PBMCs, 2 × 10^5^ cells/well) were purchased from STEMCELL Technologies (Kent, WA) and cultured in RP-10. Frozen PBMCs were thawed, washed, and rested for 1 h at 37 °C in RP-10 before stimulation with compounds. Cells were then stimulated with compound or vehicle under the same conditions as mBMDCs.

### 2.4. ELISA for the measurement of cytokines and chemokines in the culture supernatants

Cytokine levels in culture supernatants [hIL-6, hIL-8, hIL-12p70, hIFN-β and hTNF (hPBMCs) and mIL-4, mIL-5, mIL-6, mIL-12p40/p70 and mTNF (mBMDCs)] were quantified using sandwich ELISA protocols according to the manufacturer’s protocols [15]. Briefly, 96-well half-area plates were coated with capture antibodies (Table. S1) diluted in 0.1 M carbonate buffer (pH 9.6) or PBS overnight at 4 °C. Plates were washed and blocked with 1 % BSA in PBS for 2 h at 37 °C. Samples and serially diluted standards were added in incubated for 2 h at 37 °C or overnight at 4 °C. After washing, biotinylated detection antibodies and HRP-conjugated streptavidin (Thermo Fisher Scientific) were sequentially added, followed by incubation and washing. TMB substrate (KPL) was used for color development, and reactions were stopped with 1 M phosphoric acid. Absorbance was measured at 450–650 nm using a TECAN microplate reader. Standard curves were constructed using known concentrations of recombinant cytokines, and cytokine concentrations in samples were calculated accordingly. The detection ranges for all ELISA assays and the detection ranges for antibody measurements are summarized in Table. S2.

### 2.5. In vitro assays using human TLR7-SEAP HEK293

Human TLR7-secreted alkaline phosphatase (SEAP) HEK293 reporter cells were plated at 5.0 × 10^4^ cells per well in 96-well flat-bottom plates. Cells were stimulated with graded doses of test compounds dissolved in vehicle in DMEM supplemented with 10 % FBS, 1 mM sodium pyruvate, 4 mM l-glutamine, 1 % penicillin/streptomycin. Supernatants were collected after 20 h, and SEAP activity was quantified using QuantiBlue detection reagent (InvivoGen) with absorbance measured at OD_630_ using a TECAN plate reader.

### 2.6. Immunization through intramuscular and intranasal routes

Inactivated influenza A virus (IIAV) was obtained from the BEI Resource Repository. IIAV (10 μg/injection) was mixed with Lipo-**1V270** (1 nmol/injection equivalent to **1V270**) in 50 μL. MPLA (1 μg/injection) was used as a comparator. IIAV-dose response was determined by a preliminary study in Fig. S5. For intramuscular administration, 50 μL of each agent was injected into the gastrocnemius muscle using a 29G insulin syringe. For intranasal, boosting, mice were anesthetized with 2.5 % isoflurane (Fluriso^™^, VetOne) in O_2_ (flow rate 1.8 L/min) using a vaporizer and a 25 μL adjuvanted vaccine was intranasally delivered slowly to the nares using a pipetman. Immunization schedules and sample sizes are indicated in each figure legend.

### 2.7. Nasal washes

The nasal cavity was gently rinsed through the nasopharynx with 1 % BSA-PBS 300 μL using a 22G catheter and the flow-through from the nares was collected in a 1.5 mL tube [15]. Following, the supernatants were stored at −20 °C until further use.

### 2.8. Assessment for Antigen-Specific IgG and IgA antibody titers

The 96-well half-area plates were coated overnight at 4 °C with 50 μL/well of either (i) ovalbumin (OVA) at 5 μg/mL in PBS (ii) H1-HA derived from A/California/04/2009 (H1N1)pdm09; 0.1 μg/mL for IgG1, IgG2a, total IgG or 1 μg/mL for total IgA [15]. After blocking with blocking buffer, the plates were incubated with detection antibodies (Table. S1) for 2 h and washed. The plates were developed with alkalic phosphatase using a TECAN plate reader. Standard sera with known endpoint titers were used for interpolating the arbitrary unit from the standard curve. The standard sera were prepared with endpoint titers that were calculated as a reciprocal of the highest dilution that give reading double the absorbance of the background. For total IgA, diluted nasal lavage specimens were incubated overnight at RT. OD_405–570_ values were measured using a TECAN plate reader. Because IgA levels in the nasal wash were significantly lower than serum IgG levels, a narrower detection range for IgA (from 1 to 2048 arbitrary units/mL) was applied. The detailed reagents information used in Ig assessment is listed in Table. S1. The limits of detection (LOD) and the detection ranges for antibody measurements are also summarized in Table. S2. To clarify the cohort size, the animals exhibiting arbitrary titers below the LOD were plotted at the lowest values of the figures.

### 2.9. Assessment of antigen-specific splenic T-cell Responses

The splenocytes prepared using gentleMACS^™^ Octo-Dissociator (1 × 10^6^ cells/200 μL/well) were cultured with RP-10 with 10 μg/mL H1-HA. Five days later, culture supernatants were harvested and stored at −20 °C until further use. IFN-γ and IL-5 in the culture supernatants were evaluated by ELISA. Detailed information for reagents is described in Table. S1.

### 2.10. Statistical analysis

Prism 10 (GraphPad Software, San Diego, CA) was used for the generation of the dose-response curve of compounds. Kruskal–Wallis tests with Dunn’s post hoc test were applied for comparison of multiple groups. *P* values smaller than 0.05 were considered statistically significant.

## 3. Results

### 3.1. Synthesis and formulation of TLR7 ligand, 1V270

Formulations of **1V270** were necessary to deliver a homogenous dose as well as to improve cellular uptake and immune response [18]. Physical characteristics such as particle size and poly dispersity index (PDI) have been shown to influence immune response in vaccine models as well as drug delivery, and thus, sizes of <200 nm and a PDI of <0.3 were targeted [19]. **1V270** lipid nanoparticles were successfully formulated via a thin-film rehydration process and an average particle size of 74.98 ± 11.33 nm (mean ± SD) and a PDI of 0.228 ± 0.007 (mean ± SD) were observed. **1V270** was fully (100 %) incorporated into liposomes with or without additional lipids, which was confirmed by spinning the formulation at 5000 rpm to precipitate the unincorporated **1V270**, followed by quantitation of **1V270** in the resultant liposomal suspension using HPLC-MS/MS (Fig. S3). The lipid nanoparticle formulation was monitored via LC-MS for stability of **1V270** and was found to be stable for more than 6 months when stored at 2–8 °C.

### 3.2. Liposomal formulation attenuates in vitro innate immune stimulatory potency of 1V270

**Fig. 1.**
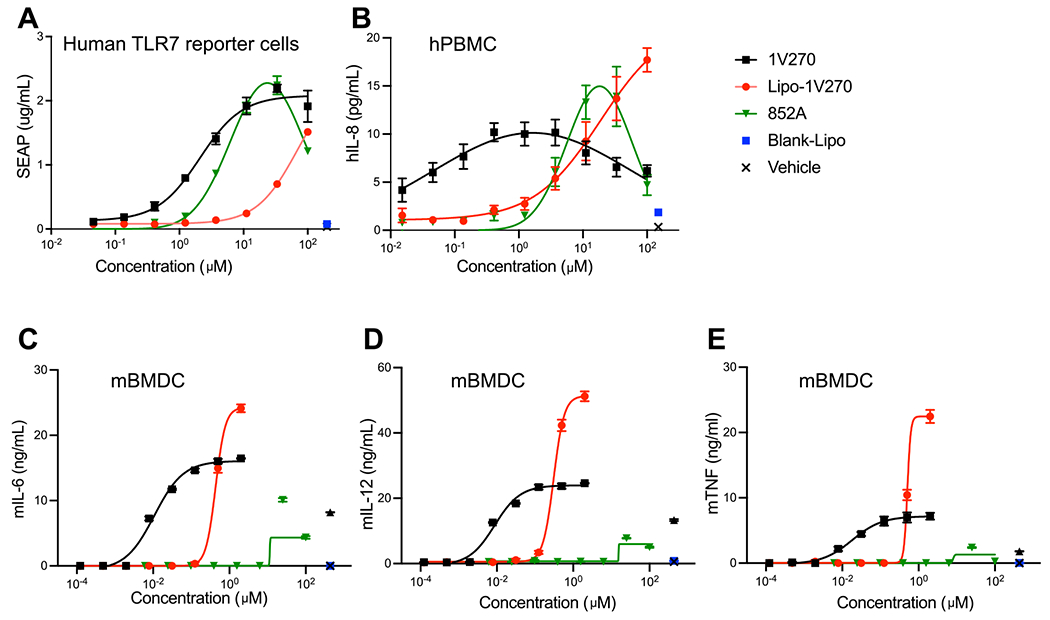
Liposomal formulation attenuates innate immune potency of 1V270. (A) Human TLR7 reporter cells (5 × 10^4^ cells/200 uL/well), (B) hPBMC (2 × 10^5^ cells/200 uL/well), and (C, D, and E) BALB/c female murine BMDC (1 × 10^5^ cells/200 uL/well) were cultured with serially diluted **1V270** and Lipo-**1V270**, and 852A. 852A is an FDA-approved TLR7 agonist and was used as a comparator. The blank liposome (Blank-Lipo) served as a positive and negative control, respectively. Blank-Lipo and vehicle were used for the negative control and LPS was used for the positive control in some experiments. After incubation, culture supernatants were collected. NF-κB inducible SEAP protein levels in the supernatants were measured by QuantiBlue reagent, hIL-8, (hPBMCs) and mIL-6, m12p40/p70, and mTNF (mBMDCs) in the culture supernatant were measured by ELISA. The data are representative of two independent experiments showing similar trends.

The lipid portion in **1V270** is easily incorporated into the lipid bilayers of liposomes, which may attenuate the pharmacophores from accessing the endosomal receptors [20]. To assess whether liposomal encapsulation alters the innate immune stimulatory potency of **1V270**, three cell types were stimulated with serial dilutions of unformulated **1V270** and Lipo-**1V270** in vitro: (1) human TLR7-SEAP HEK reporter cell line, (2) human peripheral blood mononuclear cells (hPBMC), and (3) murine bone marrow-derived dendritic cells (mBMDCs prepared from BALB/c and C57BL/6). Cytokine secretions were measured by ELISA following overnight incubation (Fig. 1 and Fig. S5). We measured the release of cytokines and chemokines, hIL-6, hIL-8, hIL-12p70, hIFN-β and hTNF (hPBMCs) and mIL-4, mIL-5, mIL-6, mIL-12p40/p70, and mTNF (mBMDCs) that are known to be released by antigen-presenting cells upon TLR7-stimulation [10,21]. Dose-dependent cytokine releases are presented in Fig. 1 and Fig. S5. In the hTLR7 reporter cell assay, unformulated **1V270** retained measurable TLR7 stimulatory activity (EC_50_ = 2.1 μM), indicating that it can directly engage TLR7 that lack endosomal uptake constraints. In contrast, mIL-12 EC_50_ values of Lipo-**1V270** were higher than those of unformulated **1V270** in all cell types [69 μM, 14 μM, and 0.3 μM (BALB/c) and 1.7 μM (C57BL/6) for hTLR4 reporter cells, hPBMC, and mBMDC, respectively, vs 2.1 μM, 0.01 μM, and 0.01 μM (BALB/c) and 0.03 μM (C57BL/6)] suggesting that liposomal formulations might restrict immediate receptor access and thereby delay ligand engagement with endosomal TLR7 [20]. hIL-6, hTNF, mIL-12, and mTNF showed similar trends (Fig. S5). Interestingly, the magnitude and pattern of responses differed between hPBMCs and mBMDC, which may reflect species-specific variations in TLR7 expression, trafficking, or downstream signaling [22,23]. Among these cytokines, hIL-12p70, hIFN-β, mIL-4 and mIL-5 were under the limits of detection (Table S2). On the other hand, the maximum inductions of cytokines by Lipo-**1V270** showed higher values than that of unformulated **1V270** by comparison in primary cells (hPBMC and mBMDC). We also compared **1V270** to **852A**, an FDA-approved dual TLR7/8 agonist, as a reference compound. Consistent with its known pharmacological profile, **852A** exhibited strong activity in a hTLR7 reporter cell assay and in hPBMCs [24,25]. In contrast, its stimulatory activity in murine BMDCs was markedly lower, with only minimal induction observed at 100 μM.

### 3.3. Lipo-1V270 enhances antigen-specific humoral responses in vivo

**Fig. 2.**
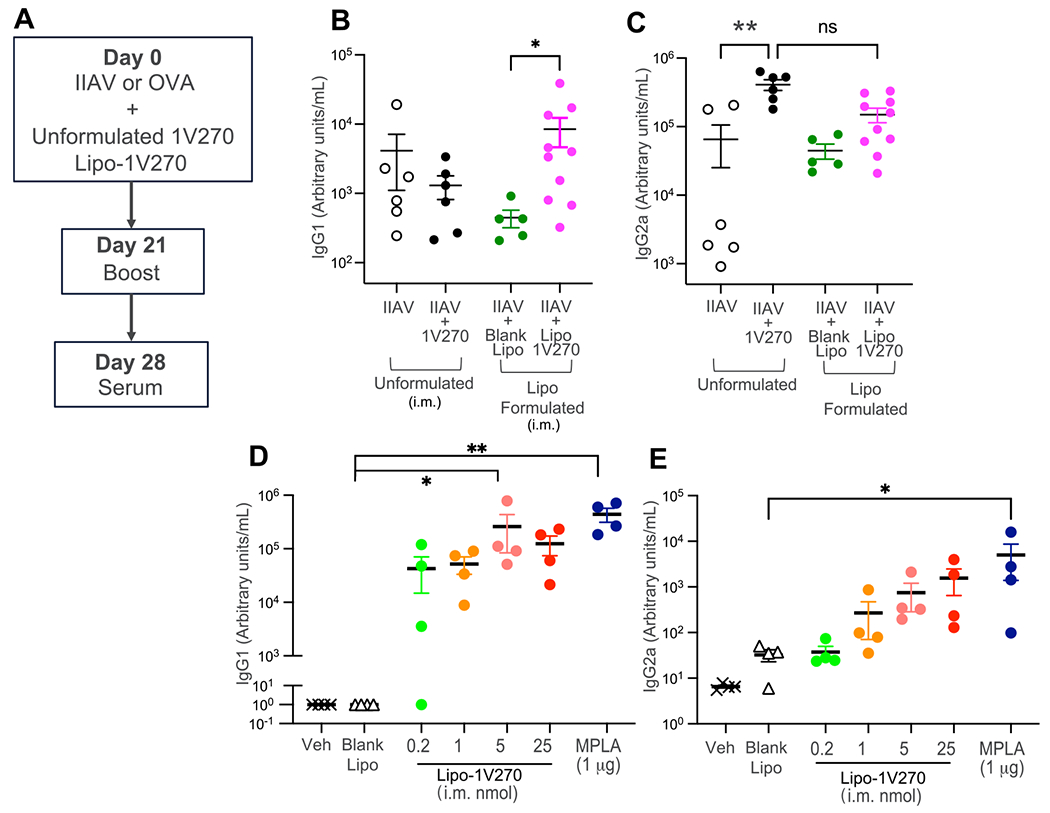
Lipo-1V270 enhances both IgG1 and IgG2a humoral responses. A) Experimental protocol. (B and C) Female BALB/c mice (6–8 week-old) (n = 4–6) were intramuscularly immunized (i.m.) with IIAV (A/California/04/2009 (H1N1)pdm09)(10 μ g equivalent to 3 μ g H1-HA/animal) adjuvanted with vehicle (10 % DMSO) unformulated 1V270 (1V270 in the figure), blank liposomes (Blank-Lipo) or Lipo-1V270 on days 0 and 21. Sera were collected on day 28 and H1-HA-specific IgG1 and IgG2a were measured by ELISA. (D and E) Mice were i.m. immunized with OVA (1 μ g/animal) adjuvanted with various doses of Lipo-1V270 (25, 5, 1, and 0.2 nmol/animal) on days 0 and 21. Sera were collected on day 28 and OVA-specific IgG1 and IgG2a were measured by ELISA. Naïve (day 0) baseline values were OVA-specific IgG1 = 332 ± 186 U/mL, IgG2a and total IgG were under the lowest detection limit. * and ** are *p* < 0.05 and *p* < 0.01. respectively, by Kruskal-Wallis followed by Dunn’s test. ns; not significant.

While in vitro data indicated reduced potency of Lipo-**1V270**, liposomes are known to enhance the immunogenicity of vaccines via depot effects and sustained release, which are best evaluated in vivo [26,27]. To investigate whether Lipo-**1V270** retains adjuvant activity in vivo, we conducted a preclinical vaccination study using A/California/04/2009 (H1N1)pdm09 and OVA as antigens (Fig. 2). Unformulated **1V270** enhanced serum IgG2a levels but failed to robustly induce H1-HA-specific IgG1 in sera [10]. In contrast, Lipo-**1V270** adjuvanted IIAV significantly increased H1-HA-specific IgG1 compared to blank liposome adjuvanted IIAV controls (*p* < 0.05, Fig. 2B and C). In OVA-immunized mice, Lipo-**1V270** induced dose-dependent antigen-specific increases in both IgG1 and IgG2a (Fig. 2D–2E). These results indicate that the liposomal formulation enhanced the adjuvant effects of **1V270** on antigen-specific humoral responses after intramuscular vaccination.

### 3.4. Intranasal administration of Lipo-1V270-adjuvanted IIAV enhances mucosal H1-HA-specific IgA and IgG in nasal washes

**Fig. 3.**
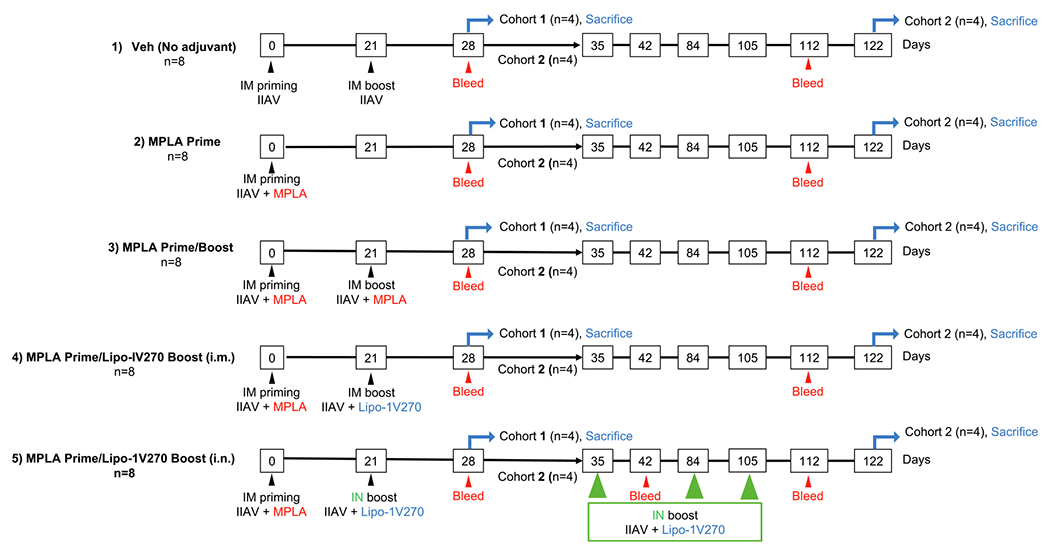
Experimental groups and schedule in heterologous prime-boost regimen. BALB/c mice were randomly allocated into five vaccination regimens (Group 1–5) and further divided into two cohort. Cohort 1 animals were euthanized on day 28 to assess early immune responses and Cohort 2 animals were euthanized on day 122 to assess long-term responses.

TLR7 is largely restricted to hematopoietic cells in humans such as pDCs and B cells, while TLR4 is broadly expressed on both immune and non-immune cells. This pattern suggests that TLR4 ligands are better suited for priming, while TLR7 ligands like **1V270** could be advantageous for boosting by targeting B-cells. Hence, we hypothesized that priming with a TLR4 agonist (MPLA) followed by intranasal boosting with Lipo-**1V270** would enhance immunogenicity of the vaccine. To test this hypothesis, BALB/c mice were primed intramuscularly with IIAV (10 μg/dose) adjuvanted with either MPLA (1 μg/dose) or vehicle. On day 21, mice were boosted with one of four regimens (Fig. 3). IIAV antigen dose was determined by a preliminary study (Fig. S4). One cohort was sacrificed on day 28, the remainder continued immunizations, and the MPLA i.m. + Lipo-**1V270** i.n. groups received three additional Lipo-**1V270** IIAV boosters on days 35, 84 and 105. Serum and nasal wash specimens were collected on days 28 and 122 to evaluate H1-HA-specific IgG1, IgG2a, and IgA. Splenocytes were also harvested to measure H1-HA-specific IL-5 and IFN-γ to assess the antigen-specific splenic responses [28].

**Fig. 4.**
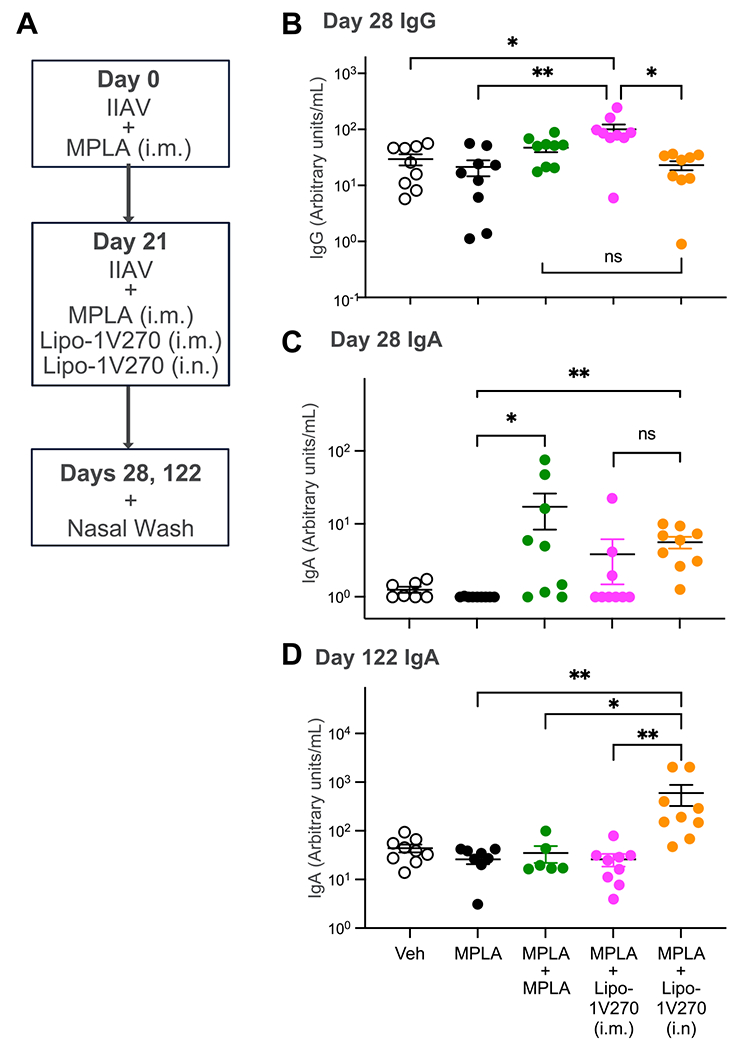
Intramuscular and intranasal Lipo-1V270 boosting induces H1-HA-specific IgG and IgA in nasal washes. Female BALB/c mice (6- to 8-week-old) (*n* = 9) were immunized on days 0 and 21 according to the immunization schedule described in Fig. 3 (**A**). On days 28 (B and C) and 122 (D), nasal washes were collected, and H1-HA-specific IgG and IgA were measured by ELISA. Naïve (day 0) baseline values were: H1-HA-specific IgG1 = 439 ± 241 U/mL, IgG2a = 2934 ± 1032 U/mL, Total IgG = 349 ± 86 U/mL* and ** are *p* < 0.05 and *p* < 0.01, respectively, by Kruskal-Wallis followed by Dunn’s test. Data were pooled from two independent experiments showing similar trends. ns; not significant.

To study whether intranasal administration of Lipo-**1V270**-adjuvanted IIAV can induce local mucosal immune responses following intramuscular priming, nasal washes were collected from the first cohort of mice on day 28 (Fig. 4A). Intranasal boosting with Lipo-**1V270**-IIAV significantly enhanced H1-HA-specific IgG in the nasal washes compared to the intramuscular MPLA-IIAV priming-only group (Fig. 4B). To evaluate whether additional intranasal boosts promote local immune responses, antigen-specific IgG and IgA were compared to day 28 (two-dose regimens) and day 122 with three additional intranasal boosts (five-dose regimen). The repeated intranasal boosting with Lipo-**1V270** significantly increased H1-HA-specific IgA in the nasal washes compared to the group that received intramuscular MPLA-IIAV priming alone (*p* < 0.01, Fig. 4C and D). Collectively, these data indicated that intranasal Lipo-**1V270** adjuvanted-IIAV effectively induced local mucosal antigen-specific IgA and IgG, demonstrating its capacity to enhance antigen-specific mucosal immunity, which is critical for protection against respiratory pathogens such as the influenza virus.

Heterologous prime-boosting regimen with intranasal Lipo-**1V270**-IIAV boosting increases systemic H1-HA-specific IgG2a comparable to intramuscular MPLA-IIAV boosting.

**Fig. 5.**
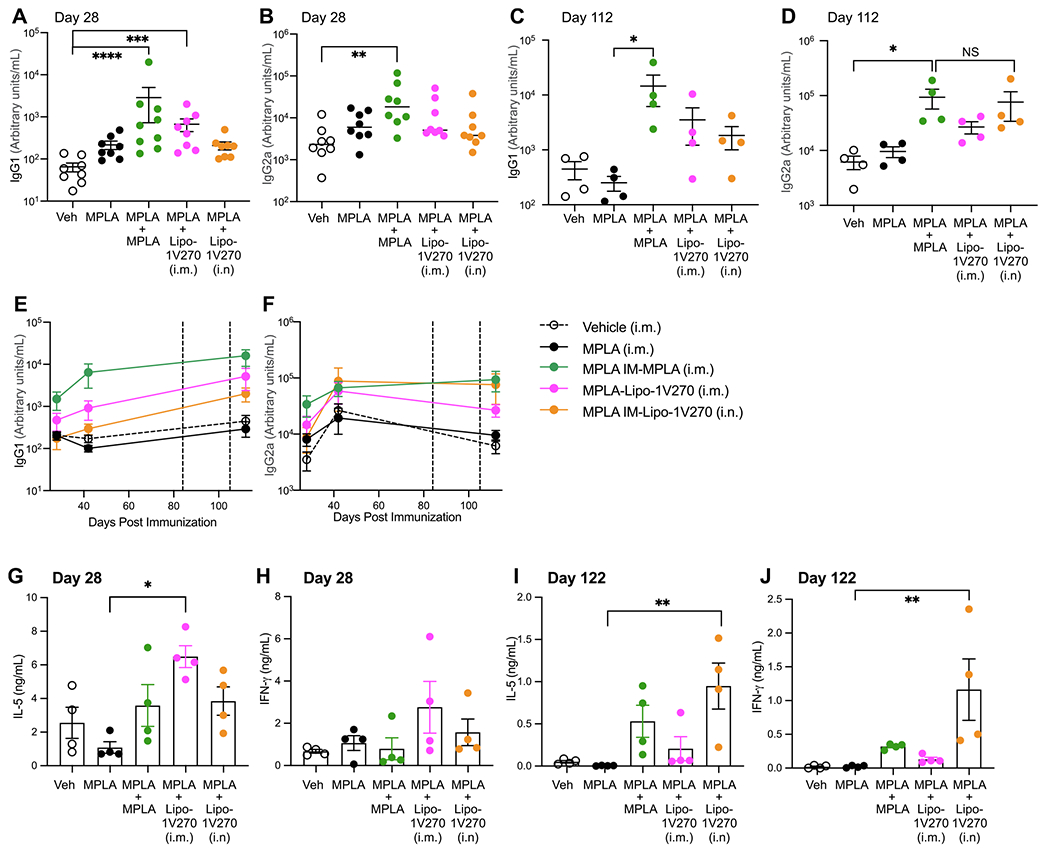
Lipo-1V270 adjuvanted IIAV vaccine induces systemic and antigen-specific IgG IgA and splenic T-cell cytokine production. (A-E) The cohorts of BALB/c mice (n = 4–8) were primed on day 0 with IIAV adjuvanted with MPLA (1 μ g/animal) or with IIAV plus vehicle. Dashed vertical lines indicate intranasal booster immunization data. On days 35, 84 and 105, the Lipo-1V270 i.n. group received an additional boost. Blood was collected on days 28, 42, and 112. H1-HA-specific IgG levels were measured by ELISA. H1-HA-specific IgG1 at day 28 (A), IgG2a (B), kinetics of IgG1 (C) and IgG2a (D) are shown. (E-H) On days 28 and 122, the cohorts of mice were sacrificed and splenocytes were harvested and cultured with H1-HA (5 μ g/mL) for 5 days. H1-HA-specific IL-5 and IFN-γ released in the culture supernatants were measured by ELISA. *, **, ***, **** and ns are p < 0.05, 0.01, 0.001, 0.0001 and not significant, respectively, by Kruskal-Wallis followed by Dunn’s test. (A-D) Data were pooled from two independent experiments showing a similar trend. (E-H) The data shown are representative of two studies showing similar results.

To examine the boosting effects of Lipo-**1V270**-IIAV on antigen-specific humoral responses, we measured serum H1-HA-specific IgG1 and IgG2a levels on day 28, one week following the boosting (Fig. 5A and B). Intramuscular boosting of Lipo-**1V270**-IIAV induced systemic antigen-specific total IgG1 at similar levels to MPLA i.m.-boosted animals on day 28 (Fig. 5A). Meanwhile, intranasal boosting of Lipo-**1V270**-IIAV had only a modest effect on systemic IgG1 release (Fig. 5A). Regarding IgG subclass on day 28, intramuscular Lipo-**1V270**-IIAV boosting induced IgG2a levels similar to those observed in the MPLA i.m. boosting group (Fig. 5B). The kinetic data showed that H1-HA-specific IgG1 antibody titers in the intramuscular MPLA boosting group were further increased (Fig. 5C). Similarly, H1-HA-specific IgG1 levels increased over time in both intramuscular and intranasal boosting groups with Lipo-**1V270**-IIAV, but the titers remained lower than those induced by MPLA-IIAV i.m. boosting (Fig. 5C). Notably, the H1-HA-specific IgG2a titers increased following intranasal Lipo-**1V270**-IIAV boosting by day 35 and was sustained through day 112 (Fig. 5D).

To assess cellular immune responses, we examined H1-HA-specific splenic T-cells by IL-5 and IFN-γ production levels which were selected as representative cytokines of Th2 and Th1 responses, respectively (Fig. 5E–H) on day 122 [28]. Mice that received multiple intranasal boosts with Lipo-**1V270**-IIAV (group 5) demonstrated antigen-specific splenic T cell responses on day 112 as evidenced by sustained IL-5 and IFN-γ production levels compared to day 28. In contrast, the splenic T-cell responses in both IL-5 and IFN-γ were decreased in MPLA i. m., MPLA i.m. + MPLA i.m., MPLA i.m. + Lipo-**1V270** i.n. on day 122 compared to day 28 (Fig. 5E-H). These findings highlighted that IIAV with an MPLA i.m.-**1V270** i.n. vaccine regimen promoted maintenance of H1-HA-splenic T-cell responses, however, this regimen was insufficient to elicit humoral responses comparable to those achieved by MPLA-IIAV administered intramuscularly.

**Fig. 6.**
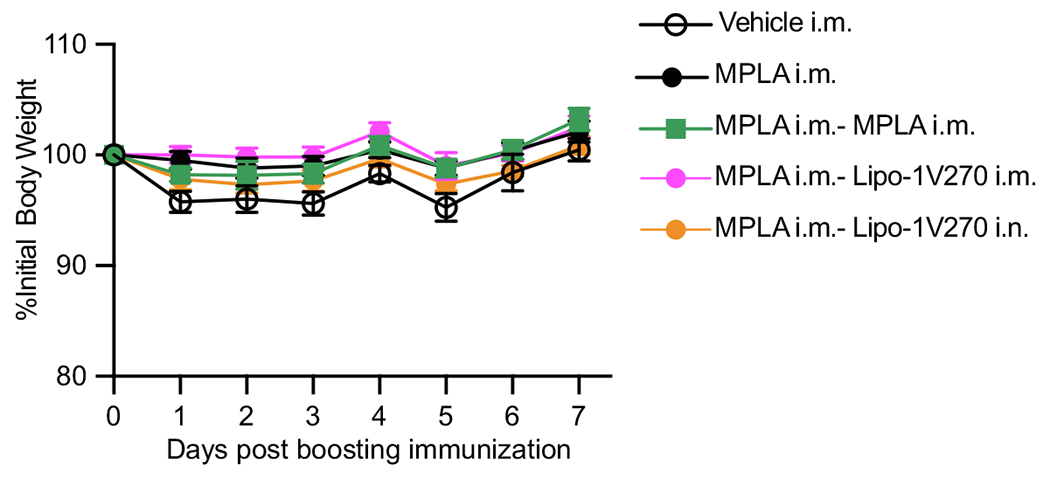
Body weight changes following vaccination. BALB/c mice (n = 4) were primed on day 0 with IIAV adjuvanted with MPLA (1 μg/animal, i.m.) or vehicle with IIAV+vehicle, and boosted on day 21 with either IIAV+MPLA i.m., Lipo-**1V270** i.m., or Lipo-**1V270** i.n. Body weights were daily monitored for 7 days following the 1st boost and normalized with preimmunization body wight. The data shown are representative of two studies showing similar results.

A non-invasive and sensitive method to evaluate immune-mediated adverse effects in murine models is essential because mice are sensitive to a broad range of stresses [29]. We also demonstrated that mice become anorexic and lose body weight following intranasal administration of TLR ligands as a result of excessive innate immune activation [30]. We, therefore, monitored body weight and observed the behaviors of mice following priming with MPLA-IIAV and subsequent boosting with Lipo-**1V270** to assess immune adverse effect (Fig. 6). No significant weight loss was observed for 7 days post-boost in animals receiving either intranasal or intramuscular Lipo-**1V270**-IIAV, indicating that both routes of immunization were well tolerated (Fig. 6).

## 4. Discussion

Multiple boosting of vaccines is widely adopted to enhance vaccine efficacy by increasing antibody titers, broadening the breadth of protection, and increasing the durability of immunity [31,32]. Currently, multiple-dose regimens are recommended for viral and bacterial infections, including Influenza, Severe acute respiratory syndrome coronavirus 2 (SARS-CoV-2), Hepatitis B, Pneumococcal conjugate, Meningococcal conjugate and the HPV vaccines. Our studies demonstrated that a heterologous prime-boost regimen, which involves priming with an IIAV adjuvant with MPLA-a component included in vaccines approved by FDA, and intranasal boosting with a liposomal formulation of **1V270**, may enhance local immune responses, suggesting its potential as a complementary strategy to conventional inactivated influenza vaccines.

Effective mucosal protection against influenza virus infection requires a dual defense mechanism, comprising both local and systemic components. Mucosal immunity is crucial for preventing virus attachment and entry, while the systemic protective immune responses reduce the severity of disease [5,33]. To address both arms of immunity, we combined intramuscular priming with MPLA that induces the systemic protective immune responses with intranasal boosting using Lipo-**1V270**, aiming to complement mucosal protection [34,35]. However, systemic H1-HA-specific IgG inductions remained suboptimal compared to homologous boosting with IIAV+MPLA. Nevertheless, our results for intranasal boosting with IIAV+Lipo-**1V270** elicited superior local H1-HA-specific total IgA in nasal wash.

Liposomal delivery is a promising strategy for mucosal vaccines and therapeutic delivery. Liposomes are biodegradable and biocompatible vesicles, which can enhance the delivery of antigens and drugs to mucosal surfaces and could protect antigens and adjuvants from degradation or disperse and enhance the activation of antigen-presenting cells residing in the local mucosal layers while reducing the systemic toxicity [36,37]. To generate an appropriate formulation for intranasal administration, we prepared a neutral liposomal formulation containing **1V270** and DOPC/cholesterol. Our in vitro experiments showed that Lipo-**1V270** increased EC50 value in the TLR7 reporter cell line, indicating the liposomal formulation reduced TLR7 agonistic potency of **1V270** (Fig. 1). This observation suggests that the components of the liposomal formulation may create a physical barrier that hinders the TLR7 ligand’s effective interaction with its receptor on immune cells [38]. Interestingly, Lipo-**1V270** demonstrated improved adjuvant effects in vivo though Lipo-**1V270** exhibited reduced TLR7-stimulatory activity compared to unformulated **1V270** in vitro. This discrepancy may be attributed to differences in receptor accessibility, while liposomal formulations could promote a controlled release of **1V270**, resulting in enhancing immunogenicity of antigens [15]. Such differences underscore the limitations of the in vitro assay. While Lipo-**1V270** presented lower potency, it still retained the immuno-stimulatory activity inducing IgG1 and IgG2a after in vivo immunization (Fig. 2).

In addition to heterologous prime-boost vaccination via distinct routes of administration, our study introduces a heterologous adjuvant regimen, combining a TLR4-based intramuscular priming (MPLA) and a TLR7-based intranasal boosting (Lipo-**1V270**). MPLA broadly stimulates myeloid cells systemically, while Lipo-**1V270** engages endosomal receptors on mucosal-resident dendritic cells and B cells. **1V270** is a TLR7-specific ligand, utilizing the adaptor protein MYD88, and failed to induce immune responses in TLR7- or MYD88-deficient mice, confirming that its immunostimulatory activity is TLR7- and MYD88-dependent [39,40]. Intranasal boosting strategy is aligned with prior efforts that use different adjuvants in prime and boost phases to broaden immune responses [33–35]. Future vaccine design could benefit from rational selection of adjuvants tailored to both delivery route and immunological targets to maximize efficacy. Interestingly, the reference compound we used in this study, **852A**, showed higher potency in hPBMC compared to mBMDC. This species-dependent difference aligns with previous reports considering interspecies variability when interpreting preclinical data for human application [24,25].

Intranasal delivery has also been clinically implemented in the form of FluMist, an intranasal influenza vaccine approved by the U.S. FDA [41]. It’s widely used as an intranasal vaccine and is particularly characterized by its strong ability to induce local IgA responses in the nasal mucosa. Intranasal administration promotes the production of IgA at the respiratory mucosal surfaces, thereby enhancing mucosal defense by preventing viral entry at the initial stage of infection. In the present study, intranasal boosting with Lipo-**1V270** significantly increased H1-HA-specific IgA levels, suggesting that Lipo-**1V270** may serve as an effective strategy for inducing mucosal immunity. Interestingly, we also observed a modest increase in nasal IgA titers in systemic vaccination groups such as MPLA+MPLA and MPLA+i.m. Lipo-**1V270** (Fig. 4). Previous studies have reported similar findings, indicating that intramuscular administration of adjuvanted vaccines can result in detectable mucosal antibody responses, albeit at lower magnitude and durability compared to mucosal vaccination [15]. These data support the concept that direct intranasal delivery, especially with repeated boosting could achieve robust and long-lasting mucosal immune responses. In our study, the pronounced IgA responses in the nasal wash, observed at day 122 were obtained in mice receiving the five-dose regimen that included three additional intranasal boosts on days 35, 84 and 105. Our results suggest that the regimen is feasible for repeated administration that provides a long-term local immune response.

The nasal cavity is physiologically equipped with defense mechanisms such as the mucus layer and mucociliary clearance, which rapidly eliminate foreign antigens and adjuvants [42]. Therefore, efficient and stable antigen delivery to the mucosal surface under such conditions requires highly optimized formulation strategies [43]. Lipo-**1V270**, with its liposomal protective structure, is designed to stably deliver both TLR7 ligands and antigens in the mucosal environment and can be evaluated as a formulation well-suited for intranasal administration. However, systemic IgG responses induced by intranasal Lipo-**1V270** were inferior to those achieved by intramuscular injection. In contrast, FluMist, being a live attenuated vaccine, possesses the ability to replicate and strongly activate innate immunity, resulting in the induction of not only mucosal but also systemic IgG responses [41]. Given that Lipo-**1V270** is a non-replicating adjuvant system based on inactivated antigens, further optimization of the formulation and immunization regimen is required to enhance systemic IgG production, including the use of additional immunostimulants or combination adjuvants.

The safety and toxicity of the vaccine are indispensable when considering the intranasal vaccine development. The nasal cavity is a unique environment that allows for the absorption of drugs into the bloodstream or transport to the brain via nose-to-brain pathways that bypass the blood–brain barrier (BBB) [1]. **1V270** showed only nonspecific weak binding to a single receptor (androgen receptor) among a panel of GPCRs, ion channels, transporters, and kinase/non-kinase enzymes suggesting lipid conjugation of **1V270** effectively reduced off-target bindings by the parent TLR7 ligands [3,13]. To further study whether the intranasal boosting immunization led to the excessive innate immune-mediated adverse effects, we monitored body weight, which is a non-invasive method commonly used to assess treatment adverse effects in murine models [44]. Mice are sensitive to physiological stress, and even minor changes in well-being can manifest as alterations in body weight [30]. In the current study, no significant changes in body weight were observed during the 7 days following intranasal boosting with Lipo-**1V270**. Given our previous report that the intranasal application of Fos47, the combination adjuvant of **1V270** plus small molecule TLR4 ligands, demonstrated a minimal immune-mediated adverse effect, our present findings suggest no inflammatory adverse effects with Lipo-**1V270** adjuvant alone, however, additional pharmacologic and immunological parameters are required for further clinical development [45].

The tissue resident memory lymphocytes are essential for maintaining mucosal immunity and protecting against pathogens that enter through mucosal surfaces, such as the respiratory tract [46]. We have demonstrated that intranasal delivery of liposomal TLR4 and TLR7 agonist combination adjuvants induces robust lung-resident CD8^+^ T cells producing IFN-γ and neutralizing antibodies, contributing to protective immunity in influenza models [15]. Building on this knowledge, the sustained mucosal antibody and splenic T cell responses observed in this study suggest the possible involvement of long-lived tissue-resident memory lymphocytes in the respiratory mucosa. Investigation of their induction and maintenance after heterologous MPLA i.m.–Lipo-**1V270** i. n. immunization will be a key direction for future mechanistic studies.

Our results indicated that intranasal Lipo-**1V270** boosts enhance a local IgA release and antigen-specific cellular immunity. However, the current regimen remains sub-optimal and requires improvement in systemic antibody protection. We will, therefore, conduct studies, e.g., neutralizing antibodies and protection by a live-virus challenge study, using good manufacturing practice (GMP) materials upon completion of optimization of the formulation of **1V270** and immunization schedule. Furthermore, there is a need to overcome significant challenges in manufacturing, supply chain management, storage complexities, and regulatory barriers in developing dual types of vaccines during the development and distribution of prime-boost vaccines that use different vaccine components, platforms, and administration routes [47,48]. Nevertheless, intranasal boosting offers the potential for repeated administration and long-term local protection, making it worthwhile to overcome the limitations of single-type vaccines.

## 5. Conclusion

Our research highlights the importance of heterologous prime-boost regimens in enhancing the protective potency of vaccines. Lipo-**1V270** for boosting immunization in mice showed minimal adverse effects, suggesting its potential suitability for repeated administration. This approach leverages the acceptable immune-mediated adverse effects and efficacy of adjuvant in FDA-approved vaccines and is complemented with intranasal administration of antigen plus Lipo-**1V270** to activate mucosal protection.

## Supplementary Material

1

## Data Availability

Data will be made available on request.

## Appendix A. Supplementary data

Supplementary data to this article can be found online at https://doi.org/10.1016/j.vaccine.2025.127778.

## Abbreviations:

BMDC: bone marrow-derived dendritic cell
DMSO: Dimethyl sulfoxide
DOPC: 1,2-dioleoyl-sn-glycero-3-phosphocholine
DOPE: 1,2-dioleoyl-sn-glycero-3-phosphoethanolamine
FDA: Food and Drug Administration
HA: hemagglutinin
HPLC-MS: High-performance liquid chromatography-mass spectrometry
i.m.: intramuscular
i.n.: intranasal
IIAV: inactivated influenza A virus
IgA: immunoglobulin A
IgG: immunoglobulin G
LC-MS: liquid chromatography–mass spectrometry
Lipo-1V270: liposomal formulation of the TLR7 ligand 1V270
LOD: Limit of detection
MPLA: monophosphoryl lipid A
PAMPs: pathogen-molecular patterns
PBMC: peripheral blood mononuclear cell
PDI: polydispersity index
pDC: plasmacytoid dendritic cell
SAR: structure-activity relationship
SEAP: secreted embryonic alkaline phosphatase
TLR: Toll-like receptor
