# Supplementary material for: Enhancing vaccine immunogenicity through heterologous prime-boost regimen with multiple nasal boosting with liposomal TLR7 ligand
Source: Vaccine. Author manuscript; Available in PMC 2026 Jun 24. (PMC13293167; doi:10.1016/j.vaccine.2025.127778)
Supplement: 1 [file NIHMS2183322-supplement-1.pdf]

Supplementary materials

Enhancing Vaccine Immunogenicity through Heterologous Prime-Boost Regimen with Multiple Nasal Boosting with Liposomal TLR7 ligand

Tomoko Hayashi [1*†], Shiyin Yao [1,] Fumi Sato-Kaneko [1], Renna Cozza [1], Hiroyuki Baba [1], Jasmine Jin [1], Ian Mclaughlin [1], Fernando Gil [1], Paola Anguiano Quiroz [1], Nikunj M. Shukla [1], Michael Chan [1], Howard B. Cottam [1], Dennis A. Carson [1*†]

1. Division of Rheumatology, Department of Medicine, University of California San Diego, 9500 Gilman Dr, La Jolla, CA 92093-0809, United States

* Corresponding author: Tomoko Hayashi

Email: thayashi@health.ucsd.edu

Table. S1. Detailed information for reagents and samples

Reagents	Source	Catalog #
OVA	Worthington biochemical (Columbus, OH)	LS003054
IIAV, A/California/04/2009/(H1N1)pdm09	BEI Resources Repository	NR-49450
1×HBSS	Corning (Union City, CA)	14175
1×PBS	Thermo Fisher Scientific (Waltham, MA)	14190
GM-CSF	BioLegend	576308
**Cell culture**		
RPMI 1640	Thermo Fisher Scientific	11875
DMEM	Thermo Fisher Scientific	11965092
Heat-inactivated dialyzed FBS	GeminiBio	100-108
Penicillin, Streptomycin	Genesee Scientific	25-512
2-mercaptoethanol	Millipore Sigma	M7522
Sodium pyruvate	Thermo Fisher Scientific	11360-070
MEM non-essential amino acids	Thermo Fisher Scientific	11140-050
Blastidin	InvivoGen	Ant-bl
MPLA	InvivoGen (San Diego, CA)	tlrl-mpls
AS01B		
**TLR-7-SEAP HEK 293 reporter cell**	**InvivoGen**	**hkb-htlr7v2**
**Cytokine ELISA kits**		
Mouse IFN-γ Duo Set	R&D systems (Minneapolis, MN)	DY485
Mouse IL-5 Duo Set	R&D systems	DY405

alpha-1 acid glycoprotein Simple Step ELISA kit	Abcam	Ab264605
Half area 96 well ELISA plate	Coring	3690
ProcartaPlex^{TM} Multiplex Immunoassay	Thermo Fisher	PPX-06-MXKA4FF
Influenzas A H1N1 (A/California/04/2009) Hemagglutinin / HA Protein (His Tag)	Sino Biological	11055-V08B
IgG1-AP goat anti-mouse	Southern Biotech	1070-04
IgG2a-AP goat anti-mouse	Southern Biotech	1080-04
IgG-AP goat anti-mouse	Southern Biotech	1030-04
IgA-AP goat anti-mouse	Southern Biotech	1040-04
p-Nitrophenyl Phosphate tablets (pNPP)	Sigma	N2770
Vaporizer	Veterinary anesthesia systems Co (Bend, OR, USA)	
Isoflurane, USP	Baxter Healthcare (Deerfield, ID, USA)	
Pipetman	Gilson Middleton (Middleton, WI, USA)	
Tecan Microplate Reader	TECAN (Männedorf, Switzerland)	30086378
Surflo ETFE Ⅳ Catherter 22G	TERUMO (Somerset, NJ, USA)	SR-OX2225CA

16

Table. S2. Limit of detection (LOD) for ELISA used in this study

**Reagents**	**Limit of detection**
Mouse IL-4	0.03-4 ng/mL
Mouse IL-5	0.03-40 ng/mL
Mouse IL-6	0.03-60 ng/mL
Mouse IL-12	0.03-60 ng/mL
Mouse IFN-γ	0.06–8 ng/mL
Mouse TNF	0.6-80 ng/mL
Human IL-8	0.3-40 ng/mL
Human IL-6	0.08-10 ng/mL
Human IL-12	0.06-4 ng/mL
Human IFN-β	0.016-1 ng/mL
Human TNF	0.03-2 ng/mL
HA IgG1-ELISA	21 ～ 23 × 10^7 U/mL
HA IgG2a-ELISA	23 ～ 21 × 10^7 U/mL
HA IgG-ELISA	42 ～ 47 × 10^7 U/mL
HA IgA-AP ELISA	1～2048 U/mL
OVA IgG1-ELISA	68 ～61×107U/mL
OVA IgG2a-ELISA	2.3 ～ 21 × 10^6 U/mL
OVA IgG-ELISA	75 ～ 67× 10^7 U/mL

Figure. S1. The synthesis of 1V270

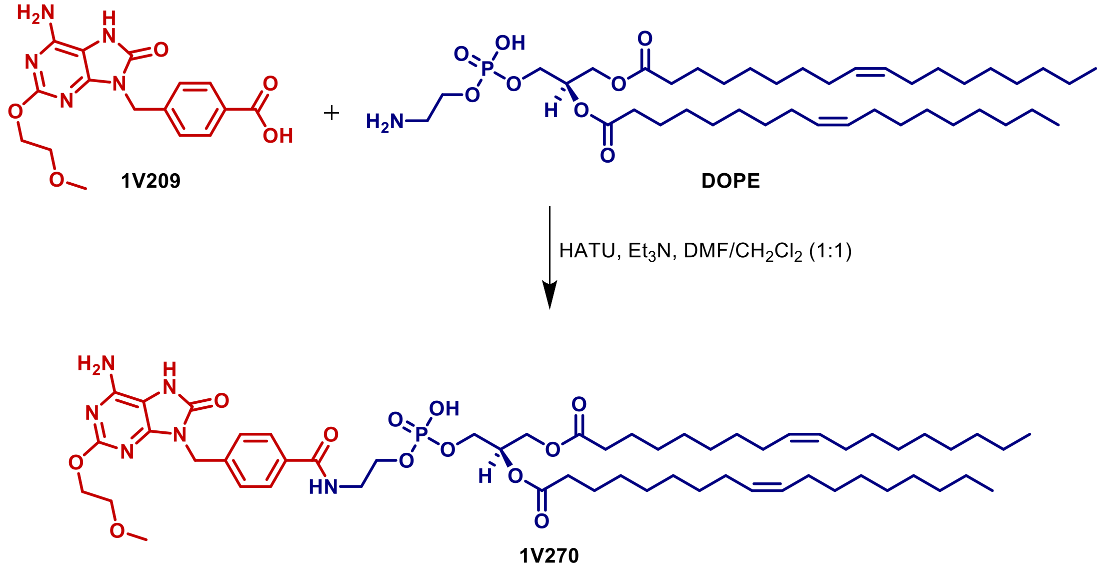

1V270 is composed of **1V209** and DOPE, which are covalently conjugated via an amide bond using Hexafluorophosphate Azabenzotriazole Tetramethyl Uronium (HATU) coupling reagent.

Figure. S2. A schematic figure describing the process of making the liposomal formulation of 1V270 (Lipo-1V270)

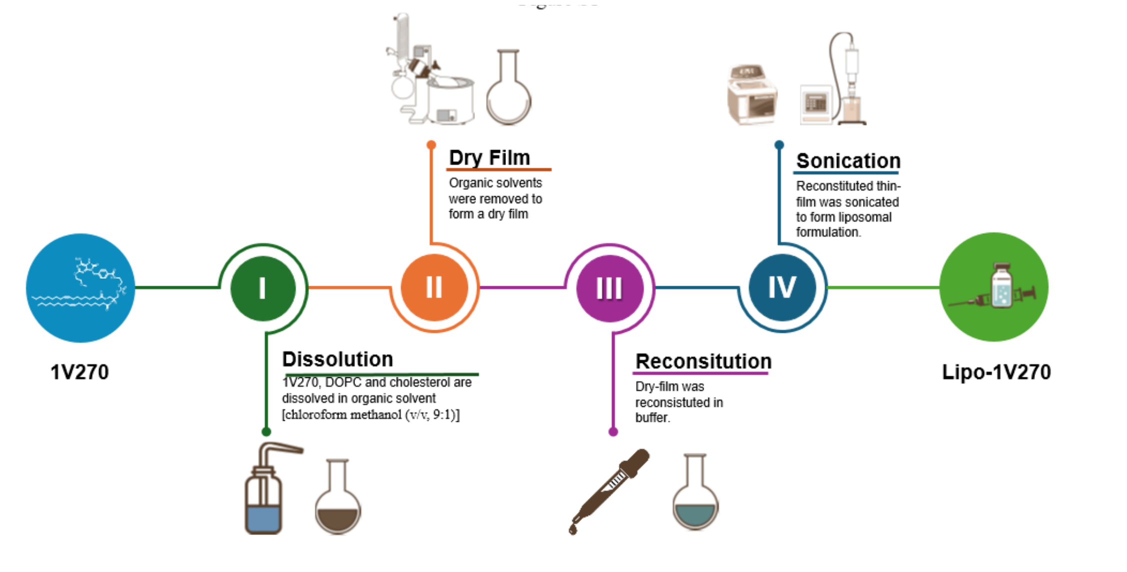

(Step I) **1V270** was dissolved in compatible organic solvents [chloroform methanol (v/v, 9:1)] with the phospholipid DOPC and cholesterol. (Step II) After ensuring all solids were dissolved, the organic solvent was removed on a rotavapor to obtain a dry film. (Step III) This dry film was then reconstituted in aqueous buffer (0.1M phosphate buffer saline pH 7.4) and (Step IV) then subjected to sonication in water bath followed by probe-ultrasonication to obtain the Lipo-**1V270**.

Figure. S3. Purity and Stability of 1V270 in Lipo-1V270

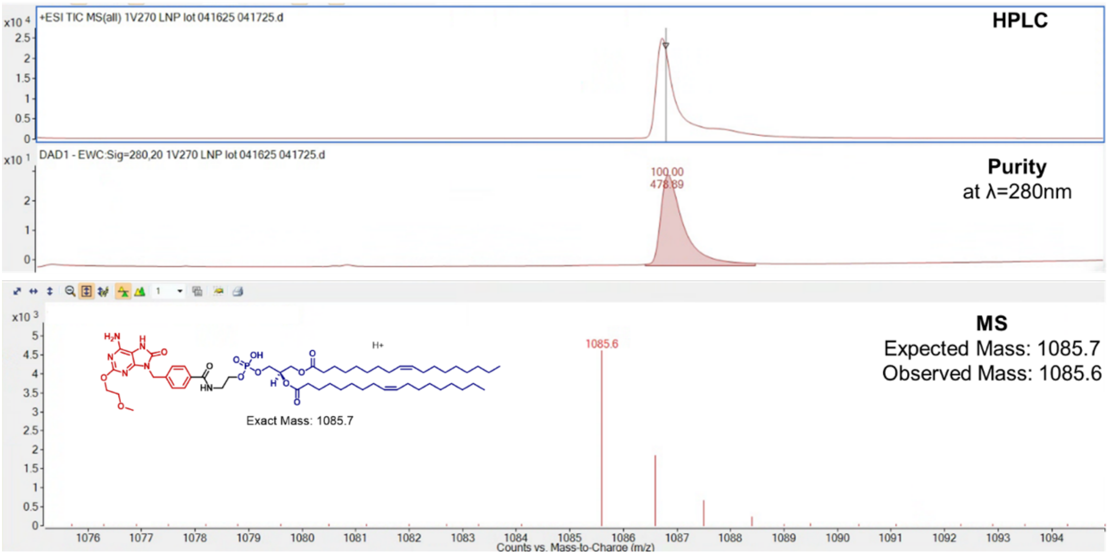

Samples were monitored for purity by HPLC-MS. Purity was judged by UV absorbance chromatogram at λ=280nm, while compound identity was verified by mass spectrometry (material and Method). More than ninety-nine % purity and 12 months (4°C) of stability were determined by HPLC-MS. HPLC-MS: High-Performance Liquid Chromatography-Mass Spectrometry

Figure. S4. Dose–response relationship of Lipo-1V270 with inactivated influenza A virus (IIAV) antigen.

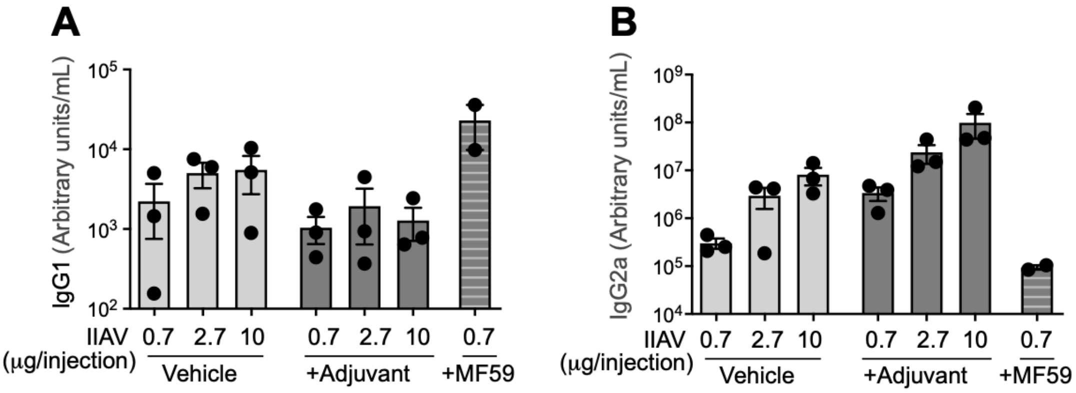

BALB/c (*n* = 3 per group) were subcutaneously immunized with various doses of IIAV mixed with 1V270 (1 nmol/injection) on days 0 and 21. Sera were collected day 28. OVA specific IgG1 and IgG2a were measured by ELISA (A and B). Data are means ± SEM.

Figure. S5. Dose–response stimulation of hPBMCs and C57BL/6-derived bone marrow dendritic cells (mBMDCs) by unformulated 1V270 and Lipo-1V270

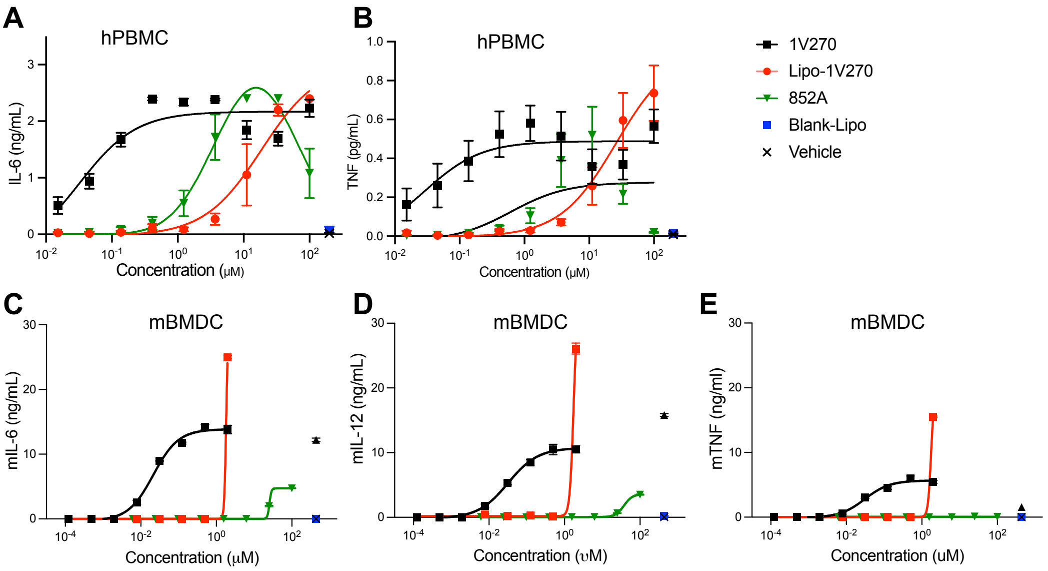

Human PBMCs (A, B) or murine BMDCs prepared from C57BL/6 mice (C, D) were stimulated in vitro with serial dilutions of unformulated 1V270, Lipo-1V270, or vehicle control for 20 h. Cytokine concentrations in culture supernatants were quantified by ELISA. Dose–response curves are shown for (A) hIL-6, (B) hTNF, (C) IL-6, (D) mIL-12p40/p70, and (E) mTNF. Data are expressed as mean ± SEM of triplicate wells and are representative of two independent experiments. Compared with unformulated 1V270, Lipo-1V270 exhibited reduced stimulatory potency, consistent with restricted receptor access upon liposomal encapsulation.
